# Supplementary material for: The interactome of intact mitochondria by cross-linking mass spectrometry provides evidence for coexisting respiratory supercomplexes
Source: Mol Cell Proteomics. 2017 Dec 8;17(2):216–32. doi: 10.1074/mcp.RA117.000470 (PMC5795388; doi:10.1074/mcp.RA117.000470)
Supplement: Supplemental Data [file supp_RA117.000470_133922_0_supp_23978_fzffwf.zip › spectra_annotation/mito_DR_spectra_annotation/148-1-1-1-11-1.pdf]

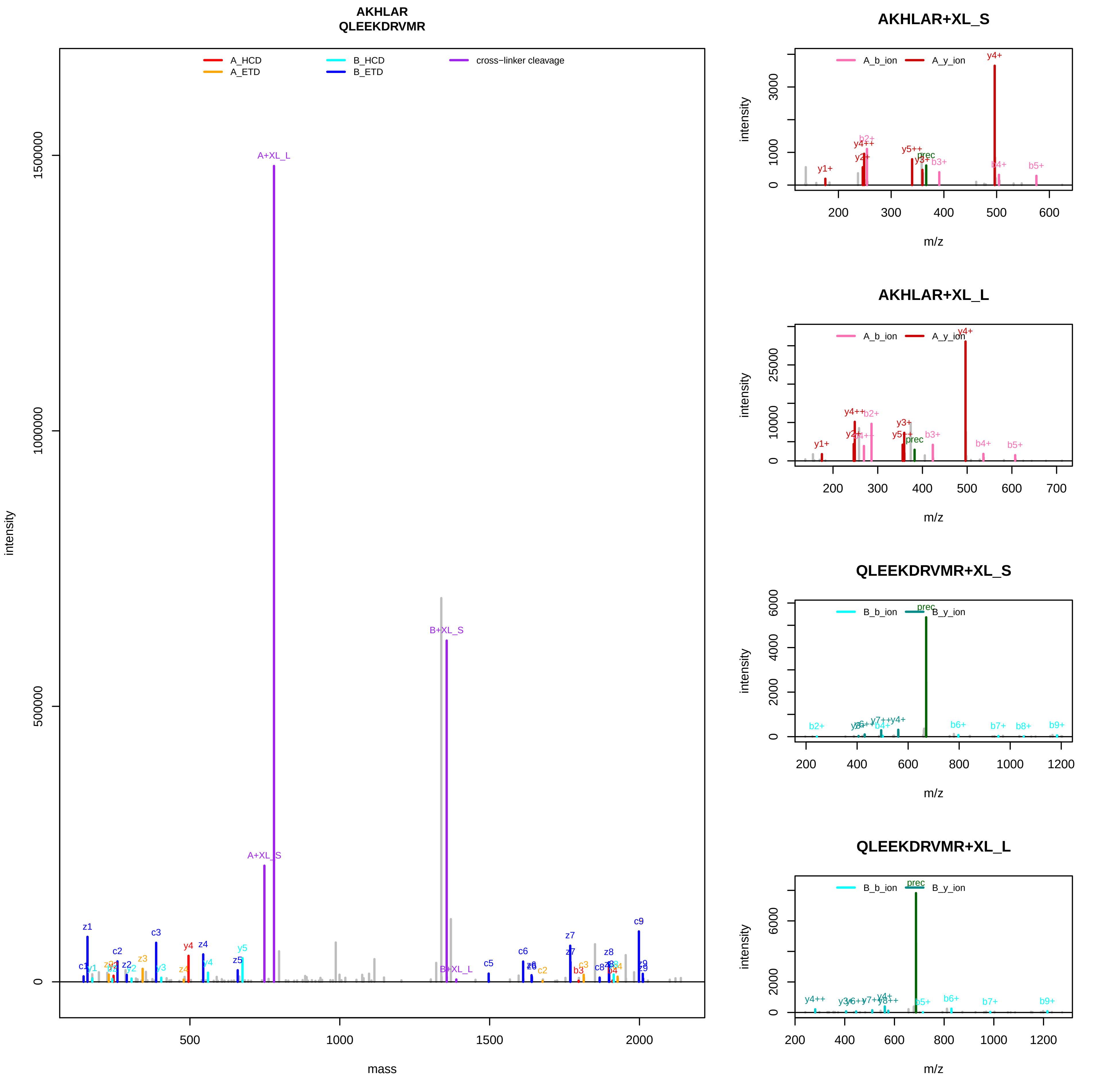

AKHLAR
QLEEKDRVMR
A_HCD
A_ETD
B_HCD
B_ETD
cross−linker cleavage
A+XL_L
B+XL_S
A+XL_S
B+XL_L
intensity
mass
0
500000
1000000
1500000
500
1000
1500
2000
z1
c1
c2
c3
z2
z3
y4
z4
y5
z5
y3
c5
c6
z7
c9
z8
c8
z9
c2
c3
b3
b4
AKHLAR+XL_S
A_b_ion
A_y_ion
y4+
y4++
b2+
y2+
y1+
y5++
prec
y3+
b3+
b4+
b5+
intensity
m/z
AKHLAR+XL_L
y4++
b2+
y3+
y5++
prec
b3+
b4+
b5+
y1+
QLEEKDRVMR+XL_S
B_b_ion
B_y_ion
prec
b2+
y7++
y4+
b6+
b7+
b8+
b9+
QLEEKDRVMR+XL_L
y4++
y4+
b5+
b6+
b7+
b9+
